# Supplementary material for: Athlete Monitoring in Rugby Union: Is Heterogeneity in Data Capture Holding Us Back?
Source: Sports (Basel). 2019 Apr 27;7(5):98. doi: 10.3390/sports7050098 (PMC6571870; doi:10.3390/sports7050098)
Supplement: Supplementary file 1 [file sports-07-00098-s001.pdf]

Premiership GPS Survey

Page 1: Introduction

To whom it concerns,

Many thanks for your continued support with training load (sRPE) and injury risk study, which is coming to the conclusion of the second season of data collection. To further understand the relationship between training load and injury risk, we have been advised by the Sports Science Advisory Group to try and collect some measure of external load to pair with the internal load already collected through sRPE. With this in mind, we are conducting this survey to establish the systems, versions, variables and definitions used across Premiership clubs when collecting and analysing GPS data for both performance measurement and injury management.

We would kindly ask that you complete the survey on the next page to help us understand the landscape of data capture across the Premiership. The results of this survey will be used by the research team for the purposes of establishing what common data elements are present across clubs. All survey responses will be kept confidential with only the research team having access to the results.

Should you have any quesions or concerns about the questionnaire, please get in touch with me at s.west@bath.ac.uk and I will do my best to answer your questions.

Many thanks as always,

Stephen West

PhD Student, University of Bath

1. What is your name? ✱ *Required*

2. What club do you work for? ✱ *Required*

3. I hereby consent to participate in the following survey, with the results being used for research purposes associated with the Professional Rugby Injury Surveillance Project. ✱ *Required*

- ○ Please tick

Page 2: Questionnaire

4. Please rate how highly you value the following measures for the the management of individual injury risk (1 being highly valued). ✱ *Required*

Please don't select more than 1 answer(s) per row.

Please select at least 11 answer(s).

	1	2	3	4	5	We dont measure this variable.
sRPE	☐	☐	☐	☐	☐	☐
GPS	☐	☐	☐	☐	☐	☐
Heart rate	☐	☐	☐	☐	☐	☐
Collision counts (Tackles, Scrums, Rucks etc)	☐	☐	☐	☐	☐	☐
Neuromuscular function (CMJ, squat jumps etc)	☐	☐	☐	☐	☐	☐
Wellbeing questionnaires	☐	☐	☐	☐	☐	☐
Other subjective ratings	☐	☐	☐	☐	☐	☐
Movement screening	☐	☐	☐	☐	☐	☐
Player age	☐	☐	☐	☐	☐	☐
Player experience	☐	☐	☐	☐	☐	☐
Previous injury history	☐	☐	☐	☐	☐	☐

5. What GPS system provider do you use? ✱ *Required*

- STATSports
- Catapult
- Other

5.a. If you selected Other, please specify:

6. What version of the GPS system do you use? ✱ *Required*

7. Have you recently (within the 16/17 season) changed provider of your GPS units? ✱ *Required*

- Yes
- No

7.a. What previous provider were you with?

8. Have you recently (within the 16/17 season) changed version of your GPS units? ✱ *Required*

- Yes
- No

8.a. What previous version were you using?

9. What model of GPS unit do you use to collect your data? ✱ Required

10. What software do you use to analyse your GPS data? ✱ Required

11. What is the measurement speed of your GPS units? (In Hz) ✱ Required

12. Which variables do you export and use from your GPS system? ✱ Required

- [ ] Total distance
- [ ] Accelerations
- [ ] Decelerations
- [ ] Distance in speed zones
- [ ] High Speed Running (distance)
- [ ] Count of Sprints

- [ ] Sprint distance
- [ ] Total Loading
- [ ] Average velocity
- [ ] Player Load
- [ ] Dynamic Stress Score
- [ ] High Metabolic Power
- [ ] Repeated High Intensity Bouts (Count)
- [ ] Repeated High Intensity Bouts (Duration)
- [ ] Peak velocity
- [ ] Meters per minute
- [ ] Tackle count
- [ ] Other

12.a. If you selected Other, please specify:

13. For training sessions, are the measurements you collect absolute (total session time) or relative (to time in play)? ✱ *Required*

- [ ] Absolute
- [ ] Relative
- [ ] Both
- [ ] Other

13.a. If you selected Other, please specify:

14. For matches, are the measurements you collect absolute (total game time) or relative (to time in play)? ✱ *Required*

- [ ] Absolute
- [ ] Relative
- [ ] Both
- [ ] Other

14.a. If you selected Other, please specify:

15. Do the variables recorded for match and training differ? ✱ *Required*

- ○ Yes
- ○ No

15.a. Which variables differ for match and training?

16. On a scale of 1-10 with 1 being the most important, how much do you value GPS data as a measure of player performance? ✱ *Required*

- ○ 1
- ○ 2
- ○ 3

- 4
- 5
- 6
- 7
- 8
- 9
- 10

16.a. Which variable do you place the greatest value on, when assessing performance? ✱ *Required*

17. On a scale of 1-10 with 1 being the most important, how much do you value GPS data to inform the management of injury risk? ✱ *Required*

- 1
- 2
- 3
- 4
- 5
- 6
- 7
- 8
- 9
- 10

17.a. Which variable do you place the greatest value on, when managing injury risk? ✱ *Required*

18. Do you use absolute (same for the whole team) or relative (individualised to players max velocity) speed bands for GPS speed zone calculations? ✱ *Required*

- Absolute
- Relative
- Both
- Other

18.a. If you selected Other, please specify:

19. What speeds are your speed zones categorised into? e.g. answer. Zone 1: 0.5-1m/s, Zone 2: 1-2m/s, Zone 3: 2-3m/s, Zone 4: 3-4m/s, Zone 5: 4-5m/s, Zone 6: 5-10m/s ✱ *Required*

20. What percentages of max velocity are your relative zones categorised into? e.g. answer. Zone 1: 0-30%, Zone 2: 30-60%, Zone 3: 60-70%, Zone 4: 70-80%, Zone 5: 80-90%, Zone 6: 90-100% ✱ *Required*

21. What speed do you classify as high speed running? ✱ *Required*

22. What speed do you class as sprinting? ✱ *Required*

23. Do you record a measure of contact (e.g. number of tackles) during training for your players? ✱ *Required*

- ◯ Yes
- ◯ No

23.a. How do you record contact events during training?

- ☐ GPS derived metrics
- ☐ Video analysis
- ☐ Other

23.a.i. If you selected Other, please specify:

24. Do you record a measure of contact (e.g. number of tackles) during matches for your players? ✱ *Required*

- Yes
- No

24.a. How do you record contact events during games?

- GPS derived metrics
- Video Analysis
- Not applicable
- Other

24.a.i. If you selected Other, please specify:

25. What sessions do you record? ✱ *Required*

- All training sessions (indoor and outdoor)
- All outdoor sessions
- Matches only
- Other

25.a. If you selected Other, please specify:

26. How many GPS units have you got access to? ✱ *Required*

27. What are the barriers to the collection of GPS data within your club? ✱ *Required*

- ☐ Lack of equipment
- ☐ No consensus on best practice for analysis
- ☐ Validity and reliability of measurement tools
- ☐ Coach "buy-in"
- ☐ Manpower required to analyse data
- ☐ Other

27.a. If you selected Other, please specify:

27.b. On a scale of 1-10 (1 being no limitation at all, 10 being highly limiting), how much do these factors limit your ability to collect and use GPS data on a day to day basis? ✱ *Required*

- 1
- 2
- 3
- 4
- 5
- 6
- 7
- 8
- 9
- 10

28. Which players do you capture GPS for? ✱ *Required*

- All players
- All senior squad players
- Key players only
- Other

28.a. If you selected Other, please specify:

Page 3: Thank you

Many thanks for completing the survey for us. If you have any questions or would like to know more about the study, please get in touch with me at s.west@bath.ac.uk
